# From intestine to beyond: *Salmonella* entry factors display distinct transcription pattern upon infection in murine models

**DOI:** 10.1098/rsob.230312

**Published:** 2024-01-17

**Authors:** Michaël Koczerka, Isabelle Lantier, Marie Morillon, Justine Deperne, Camille D. Clamagirand, Isabelle Virlogeux-Payant, Olivier Grépinet

**Affiliations:** INRAE, Université de Tours, ISP, 37380, Nouzilly, France

**Keywords:** *Salmonella*, invasion, T3SS-1, invasins, murine models, *in vivo* imaging

## Abstract

The infectious process of bacteria of the genus *Salmonella* requires the finely regulated use of various virulence factors. Among them, the type 3 secretion system-1 (T3SS-1) and the Rck and PagN invasins are involved in the internalization of the pathogen within eukaryotic cells, but their precise role in the host and in the pathogenic process is still poorly understood. In this study, we aimed to determine the kinetics of expression of these entry factors in a typhoid fever-like and a gastroenteritis model in mice by *in vivo* imaging using bioluminescent *Salmonella* Typhimurium reporter strains carrying chromosomal transcriptional fusions. Only *pagN* and T3SS-1 transcription has been clearly identified. Independently of the pathological model, the caecum was identified as the main transcription site of both *pagN* and the T3SS-1-encoding gene both at early and late stages of the infection. An intense transcription of *pagN* was also observed in deep organs in the typhoid fever-like model, while that of T3SS-1 remained quite sporadic in these organs, and mainly focused on the intestine all along the infection. This work will help to understand the respective role of these entry factors at the cellular level in the pathogenesis of *Salmonella in vivo*.

## Graphical abstract

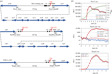

## 1. Introduction

*Salmonella* are among the most prevalent foodborne pathogens, being notably able to infect a wide variety of species ranging from plants to animals, including humans. They can be responsible, depending on the infected host, the infecting serovar and their set of virulence factors, for various pathologies such as gastroenteritis or systemic diseases including typhoid fever. In 2017, these bacteria—mainly those of the *enterica* subspecies—were responsible for 95.1 million cases of human gastroenteritis worldwide and led to the death of 50 771 individuals [1]. Typhoid and paratyphoid fevers, caused by the human-restricted serovars *S.* Typhi, *S.* Sendai or *S.* Paratyphi A, B and C, affected on the other hand 11.8 million people in 2016, and caused 128 200 deaths mainly in developing countries [2].

As an enteric pathogen, *Salmonella* is mainly orally transmitted, following the ingestion of contaminated water or food. After passing through the stomach, the pathogen reaches the intestine and interacts with the intestinal epithelium to continue its pathogenic cycle. Adhesion to and invasion of the cells of this epithelium are important steps for intestinal colonization and crossing of the epithelial barrier. *Salmonella* can either be captured by phagocytic cells (e.g. dendritic cells) or M cells, or induce its own internalization within non-phagocytic cells such as enterocytes [3]. Once in the lamina propria, the bacteria will be captured by resident phagocytic cells, where its fate will differ depending on the virulence of the strain towards the infected host. In the case of localized infections, dissemination will be stopped at the level of the mesenteric lymph nodes (MLN) while an acute, pathogen-induced inflammatory response will develop within the intestine, leading to oedema formation and fluid secretion. Conversely, serovars capable of carrying out systemic infections will limit the inflammatory response and the damage caused to the intestinal epithelium. They are captured by the macrophages underlying the epithelium and use these cells as a vector for their dissemination to the MLN, the bloodstream and ultimately systemic organs such as the spleen and liver. Following the acute stages of the infection, *Salmonella* can persist asymptomatically in organs such as the gallbladder, acting as a pathogen reservoir fueling its excretion in the environment [4].

In any case, host cell invasion remains a key step in *Salmonella* pathogenesis. To this end, the pathogen can employ a subset of virulence factors, termed entry or invasion factors, namely the T3SS-1 (Type 3 secretion system no. 1) and the PagN and Rck invasins [5]. The T3SS-1 consists of a multi-molecular complex assembled in a needle-like structure. It translocates bacterial effectors within the eukaryotic cell cytosol, thus hijacking the host-cell signallization pathways and manipulating the cytoskeleton to promote bacterial internalization through a process qualified as a Trigger mechanism [5,6]. Some of these effectors are also involved in the modulation of the inflammatory response by interfering with different pathways (e.g. NF-kB, JNK, etc.) [7], as well as in the formation and early maturation of the *Salmonella*-containing vacuole (SCV). The outer membrane proteins (OMPs) Rck and PagN also mobilize regulators of the cytoskeleton dynamics, but this mobilization occurs following the interaction of the invasins with an eukaryotic receptor, the epidermal growth factor receptor (EGFR) and the heparinated proteoglycans for Rck and PagN, respectively [8,9]. Although the targeted pathways are similar to those involved in the Trigger internalization mechanism (e.g. Arp2/3 complex, PI3K pathway), the membrane rearrangements induced following the invasin/receptor interaction are weaker, and the mechanisms involved are similar to those observed during a Zipper invasion mechanism [10,11].

Each invasion factor is subject to very distinct regulatory mechanisms. Regulation of T3SS-1 expression is quite complex and has been extensively studied as elegantly reviewed by Lou *et al.* [12]. It integrates numerous pleiotropic regulators, such as the two-component regulatory systems BarA/SirA [13] and EnvZ/OmpR [14] or the nucleoproteins H-NS [15] and Hha [16]. Once activated or repressed in response to environmental stimuli (e.g. the availability of iron or magnesium, osmolarity, pH, etc.), this pattern modulates the activity of HilA, the central transcriptional regulator of T3SS-1 expression, which ultimately leads to the expression of the system within the host's gut [17–19].

On the other hand, little is known regarding the expression of the PagN protein, except that it depends on a restrictive environment such as a moderate acidic pH, a deprivation of calcium or magnesium ions, or the presence of anti-microbial peptides, which activates the PhoP/PhoQ two-component system [20,21]. While PhoP/PhoQ is primarily described to be active within intravacuolar *Salmonella*, several reports also suggest a role of this two-component system within the gut lumen [22,23]. Moreover, little is known about the expression of PagN *in vivo*. The deletion of the *pagN* gene leads to a decrease in the virulence of *Salmonella* compared to a parental strain. This attenuation is reflected by a reduction in the clinical signs of infection (inflammatory state, degradation of the intestinal epithelium, etc.) as well as by an increase in the survival rate of infected animals, thus demonstrating its importance *in vivo* [24]. However, the precise conditions and sites of PagN expression *in vivo*, as well as the cells targeted by this invasin remain to be determined.

Similarly, the mechanisms underlying the expression of the virulence plasmid-encoded *rck* ORF, carried by the *pefI-srgC* operon are poorly understood [25]. Two promoters have been identified upstream of the *pefI-srgC* operon [26]. While the activation of one of them is dependent on the quorum-sensing regulator SdiA, itself responsive to the presence of N-Acyl homoserine lactone (AHLs) in the environment, the other responds to parameters which remain to be identified [26–28]. Its *in vivo* expression profile remains to be determined as well, since to date and despite numerous studies, no activity of the quorum sensing regulator SdiA or of the SdiA-dependent promoter region has been identified in mouse models of *Salmonella* infection except in the presence of AHL-producing bacteria [29].

An important question that remains to be elucidated is why *Salmonella* uses several invasion factors to enter eukaryotic cells. Several hypotheses can be put forward. Invasion factors could allow *Salmonella* to enter different cell types, serve in different organs and/or be involved in cell invasion at different time points in the kinetic of host infection according to the environmental conditions encountered by the bacteria. In order to provide information on this topic, we sought to determine the *in vivo* kinetics of transcription of each entry factor using *S*. *enterica* subsp. *enterica* serovar Typhimurium (hereafter named *S.* Typhimurium), one of the serovars for which the presence of the three entry factors has been described [30]. To this end, we designed chromosomally-encoded transcriptional fusions harbouring the promoter-less *luxCDABE* operon (of *Photorhabdus luminescens*) and the promoter regions of the genes encoding T3SS-1-structural proteins (*inv/spa* operon), the invasins Rck or PagN and used *in vivo* imaging to follow the transcription of each entry factor in a gastroenteritis and a systemic infection (typhoid-like) murine models.

## 2. Results

### 2.1. Validation of bioluminescent fusions reporting entry factors transcription

To identify the potential sites of expression of the different entry factors, we took advantage of the luminescent properties conferred by the *luxCDABE* operon of *P. luminescens* to design luminescent transcriptional fusions and assessed them *in vivo* during infection in commonly used salmonellosis murine models. Before animal experiments, luminescent fusions were first constructed and inserted in the unique *att*Tn7 sequence on *Salmonella* chromosome (electronic supplementary material, figure S1) and then validated *in vitro* according to the literature.

**Figure 1.**
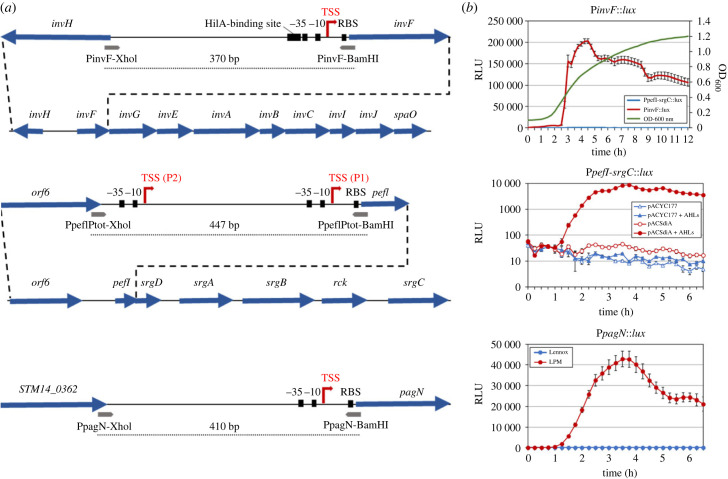
Construction and validation of the chromosomal transcriptional fusions. (*a*) Schematic representation of the promoter regions selected for the construction of transcriptional fusions. The promoter regions were selected to contain the transcriptional start sites (TSS, red broken arrows) and known or putative regulatory regions of each gene/operon of interest. From top to bottom, promoter regions of *invF*, *pefI-srgC* and *pagN* are represented. The *pefI-srgC* operon bearing the *rck* ORF and part of the *inv/spa* operon are also schematized under their promoter regions. ORFs are indicated by blue arrows, while the hybridization sites of the primers used for the amplification are represented below the ORFs. The RBS (ribosome binding site) and the -10 and -35 regions are represented by black squares. The dotted lines correspond to the promoter regions cloned in the transcriptional fusions. (*b*) Validation of the transcriptional fusions. The *S*. Typhimurium strains harbouring the chromosomal transcriptional fusions were grown in media or conditions known to induce their respective promoters (see Material and methods section). Kinetic bioluminescence signals and optical density were measured with 15-min intervals. Graphs represent the relative luminescent units (RLU or luminescence/OD_600_) as a function of time. For the P*invF*::*lux* fusion, the P*pefI-srgC*::*lux* fusion was used as a negative control and OD_600_ was also represented on the graph. For the P*pefI-srgC*::*lux* fusion, the *y*-axis is in log scale to facilitate the visualization of the various controls. Average values (± standard error of the mean) of RLU were calculated based on at least three independent assays.

Most of the T3SS-1 components and associated proteins are encoded on a pathogenicity island, of approximately 40 kb, named SPI-1 and conserved between the different clades composing the genus [31]. SPI-1 is organized in operons, which include genes encoding structural proteins, effectors, chaperone proteins but also regulatory proteins. Among them, the *inv/spa* operon represents a marker of choice to study the kinetics of T3SS-1 expression. Directly regulated by the central regulator HilA, *inv/spa* encodes many structural components of the apparatus but also the InvF regulatory protein, involved in regulating the transcription of numerous secreted effector-encoding genes. *In vitro*, the transcription of the *inv/spa* operon was shown to be dependent on the growth phase [32]. In order to monitor the kinetics of SPI-1 transcription, we cloned the intergenic region between the *invH* and *invF* ORFs, including the P*invF* promoter governing the transcription of the *inv/spa* operon, in front of the *luxCDABE* operon (figure 1*a*). Kinetic measurements of the P*invF*::*lux* reporter fusion showed no induction up to OD_600_ equal to 0.3 (MEP: mid exponential growth phase) whereas a strong induction was observed between OD_600_ 0.3 and 1.0 (LEP: late exponential growth phase) (figure 1*b*). Maximum value reached 202 980 RLU at the end of the exponential phase. The P*pefI-srgC*::*lux* fusion used as a negative control showed as expected no induction regardless of the growth phase in this culture condition (figure 1*b*) and the strain harbouring the constitutive sig70c35::*lux* fusion (used as a positive control) emitted a strong luminescent signal from the beginning of the culture (OD_600_ < 0.1) (data not shown). These results agree with those obtained by Kröger *et al.* [32] and demonstrate the functionality of the P*invF*::*lux* fusion.

The transcription of the *pefI-srgC* operon (encoding Rck) was monitored by cloning the entire intergenic region between *orf6*, belonging to the *pef* operon located just upstream of the *pefI-srgC* operon, and the *pefI* ORF, in order to encompass the two promoters already identified upstream of the *pefI-srgC* operon and putative regulatory regions (figure 1*a*) [26]. While the regulatory mechanisms governing the activity of the proximal promoter remain to be identified, previous studies described the dependence of the distal one on the quorum sensing regulator SdiA, itself activated in response to the presence of AHLs within the culture environment [27]. The P*pefI-srgC*::*lux* transcriptional fusion was therefore validated using a plasmid constitutively expressing SdiA (pACSdiA) and these specific culture conditions (figure 1*b*). In the presence of the empty vector (pACYC177), no bioluminescent signals were observed, in absence or presence of AHLs. On the contrary, addition of 2 µM AHLs to the culture medium of the strain overexpressing SdiA allowed the detection of bioluminescent signals reporting P*pefI-srgC* activity, thus validating this transcriptional fusion. The highest value (8740 RLU) for the P*pefI-srgC*::*lux* fusion was measured after 3.75 h of culture corresponding to the end of the exponential phase, and was much lower than that of the P*invF*::*lux* fusion.

Unlike the *inv/spa* and *pefI-srgC* operons, the *pagN* gene is transcribed as a monocistron. To be as exhaustive as possible on the potential regulatory mechanisms involved in its expression, we integrated into the *lux* transcriptional fusion the whole intergenic region separating *pagN* from the STM14_0362 gene (figure 1*a*). PagN expression is known to depend on the PhoP/PhoQ two-component regulatory system. *In vitro*, *pagN* transcription is induced in culture medium mimicking PhoP/PhoQ activating conditions, i.e. acidic pH and low divalent cations concentrations (LPM medium) [21,32]. As seen in figure 1*b*, bioluminescent signals could be detected after 90 min of culture in LPM medium while no signals could be measured in Lennox broth (control medium), which confirmed the functionality of the P*pagN*::*lux* fusion. Maximum value reached 42 830 RLU after 3.5 h of culture, which is intermediate between the P*pefI-srgC*::*lux* and the P*invF*::*lux* fusions. Finally, in all the culture conditions tested to validate the three *lux* fusions, no signals were measured with the mock strain 14028 *att*Tn7-NoP::*lux* which carries a promoterless *luxCDABE* operon (data not shown). This absence of non-specific transcriptional signals at the *att*Tn7 site therefore confirms the compatibility of *att*Tn7 as an integration site for transcriptional fusions as described by Shivak *et al.* [33]. Collectively, these experiments validate the use of our *lux* transcriptional fusions as accurate transcriptional reporters of the SPI-1 *inv/spa* operon, the *pefI-srgC* operon and the *pagN* gene for subsequent *in vivo* experiments.

### 2.2. Determination of basal bioluminescent intensity levels of isolated organs

Usually, bioluminescence experiments, aiming to study gene transcription *in vivo,* use non-inoculated mice as negative controls. In order to determine the true ‘basal’ bioluminescence emitted by each targeted organ, the strain 14028 *att*Tn7-NoP::*lux* was used in our mice experiments as a negative control and the radiance was measured for each organ at all time points during each experiment (29 measurements for each organ). Using these conditions, the basal radiance averages around 500 p s^−1^ cm^−2^ sr^−1^ for each organ, and according to these measurements, we arbitrarily set up a positivity threshold (PT) of the bioluminescent signals to $PT=1.5x(\mathrm{Mean}\;{\mathrm{radiance}}_{NoP}^{\mathrm{Organ}}+SD)$ (electronic supplementary material, figure S2). Any signal above this threshold was considered in the rest of this article as a signal reflecting an activity of the luciferase system and, consequently, of the studied promoters.

### 2.3. Transcription of the *inv/spa* operon is essentially detected in the distal small intestine and in the caecum

**Figure 2.**
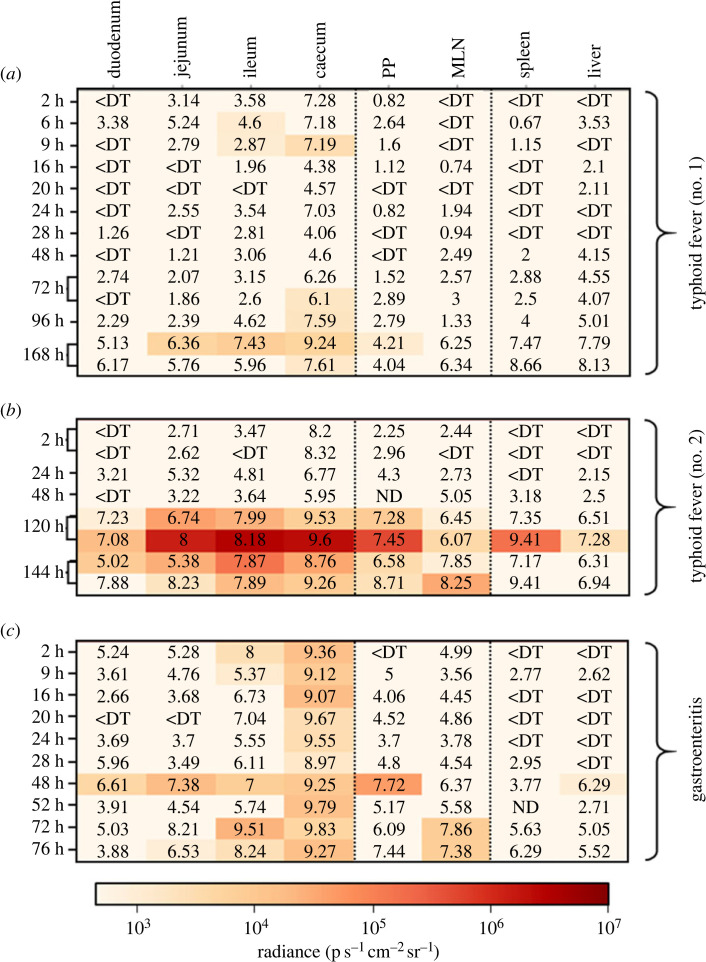
Absolute intensities of bioluminescent signals associated with *inv/spa* transcription. Heat maps representing the luminescence intensities measured in each of the target organs in mouse models (*a* and *b*) of typhoid fever (BALB/c line) or (*c*) of gastroenteritis (BALB/c line pretreated with streptomycin) following oral inoculation of the animals with 5 × 10^8^ CFU of *S*. Typhimurium 14028 P*invF*::*lux* strain. The observation times post-infection are specified on the left of the maps, and the bacterial loads expressed in log CFU per gram are indicated on the maps. For the PP, the bacterial loads are expressed in log CFU per PP. ND means not determined and <DT corresponds to organs containing no *Salmonella* or a bacterial load below the detection threshold. PP: ileal Peyer's patches; MLN: mesenteric lymph nodes.

Literature reports agree that the optimal conditions for the expression of the T3SS-1 are found within the intestinal lumen [19,34,35]. However, the exact timing of this induction, the putative expression in other organs and the differential expression profile of the secretion system depending on the pathology have not been described yet. In the typhoid fever model, we were able to detect P*invF* promoter's activity within the ileum 6 h and 9 h post-infection, and within the caecum 9 h post-infection (figure 2*a*). It might be important to raise that comparable bacterial loads were measured at the same time post-infection within other organs (e.g. jejunum) without bioluminescent signals being detected there. These results show that the activity detected for our promoter of interest is not strictly dependent on the bacterial load measured but might be the result of infection-dependent environmental changes favoring the transcription of the *inv/spa* operon within the ileum and caecum. No signals could be detected within the animals evaluated between 16 h and 72 h post-infection, in which one could note an overall decrease in the intestinal bacterial load potentially limiting our detection capacity (figure 2*a,b*). This decrease of bacterial load is probably due to the elimination of most of the inoculum by the animals at these early time points after *S.* Typhimurium oral inoculation. Nevertheless, the absence of bioluminescent signal in the caecum of the mouse analysed at 24 h post-infection which carried approximately 7 log10 CFU g^−1^, suggests that the environmental conditions encountered by *Salmonella* in the caecum of this animal at this time point are not favorable for P*invF* transcription.

Whole mouse body imaging 72 h post-infection suggested some variability in the intensity of P*invF* activity from one animal to another (data not shown). This result prompted us to assess the activity of our promoter of interest on organs isolated from two different animals. In the first one, no signals could be detected within any of the organs selected for this study while the caecum of the second one emitted bioluminescent signals, despite comparable caecal bacterial loads between both animals, thus confirming a heterogeneity of *inv/spa* operon transcription among animals. The caecal activity detected 72 h post-infection in the positive mouse is in line with the P*invF* promoter activity observed 24 h later (96 h post-infection), suggesting that changes in the intestinal environment favouring the transcription of the operon occur as the infection progresses (figure 2*a*). At later time points (120 h to 168 h post-infection) (figure 2*a*,*b*), bioluminescent signals were detected all along the small intestine, i.e. in the duodenum, jejunum, ileum, ileal Peyer patches and caecum, but also in the MLN of two animals (figure 2*a*,*b*). Surprisingly, signals reflecting *inv/spa* transcription were also detected within the spleen and the liver of one highly colonized animal autopsied 120 h post-infection, thus supporting these systemic organs could, in some cases, represent an environment conducive to T3SS-1 expression.

**Figure 3.**
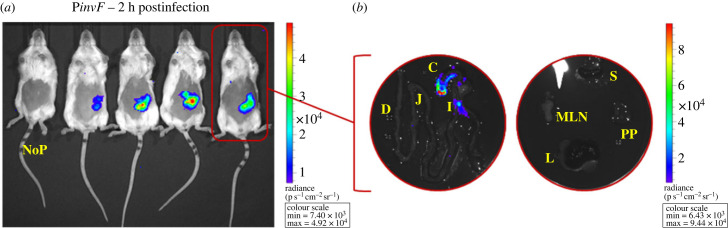
*In vivo* imaging of infection with a *Salmonella* strain expressing the *lux* operon under the control of the P*invF* promoter in a murine gastroenteritis model. BALB/c mice pretreated with streptomycin were orally inoculated with 5 × 10^8^ CFU of *S*. Typhimurium 14028 carrying the mock fusion (no promoter: NoP::*lux*) or the P*invF*::*lux* transcriptional fusion. (*a*) One control mouse (NoP) and four P*invF*::*lux* mice were imaged at 2 h post-infection with the IVIS Spectrum. The colour scale shows relative signal intensity or radiance (expressed in p s^−^^1^ cm^−^^2^ sr^−^^1^), red being the most intense and blue the least intense. (*b*) Isolated intestine and organs from one P*invF*::*lux* mouse were imaged at 2 h post-infection. C, caecum; D, duodenum; I, ileum; J, jejunum; L, liver; MLN, mesenteric lymph nodes; PP, ileal Peyer's patches; S, spleen.

To further explore the impact of variation in the intestinal environment on the activity of the P*invF* promoter, a kinetic experiment was carried out in the streptomycin pre-treated mice model developed by Hardt Lab [36]. This model enables *Salmonella*-induced gastroenteritis to be studied, reproducing notably the intestinal inflammation phenomenon. In this gastroenteritis model, we were able to observe an exacerbation of the signals associated with the transcription of the *inv/spa* operon within the digestive tract (figures 2*c* and 3), as they could be detected as early as 2 h post-infection in the caecum and the ileum, reflecting the highest contamination level of these organs and/or a more intense and an earlier transcription of *inv/spa* in the gastroenteritis model compared to the typhoid-like model. The signal intensities measured in the ileum of mice autopsied 9 h post-infection decrease below our positivity threshold, independently of the measured bacterial load. Indeed, the bacterial loads in the ileums of the animals autopsied at 16 h, 20 h, 24 h and 28 h post-infection were similar or even higher than that measured on the animal autopsied at 9 h post-infection, suggesting that environmental changes, favourable to the transcription of the *inv/spa* operon, occur during infection within this organ. Bioluminescent signals were also detected at some time points from 48 h post-infection in the ileal Peyer's Patches, the MLN or the liver. As previously observed in the typhoid model, these signals were observed only in highly contaminated mice.

Altogether, these results demonstrate (i) that the *inv/spa* operon and consequently most probably the T3SS-1 encoding genes of *S.* Typhimurium can be transcribed all along the small intestine, (ii) that differences among individuals can be observed and (iii) that the caecum is the intestinal part where *inv/spa* is most commonly transcribed and/or easily detected. Moreover, they show that some environmental conditions encountered in the MLN, the spleen and the liver can favour the transcription of this operon.

### 2.4. P*pefI-srgC* activity was only detected in the caecum of mice at a very weak level

**Figure 4.**
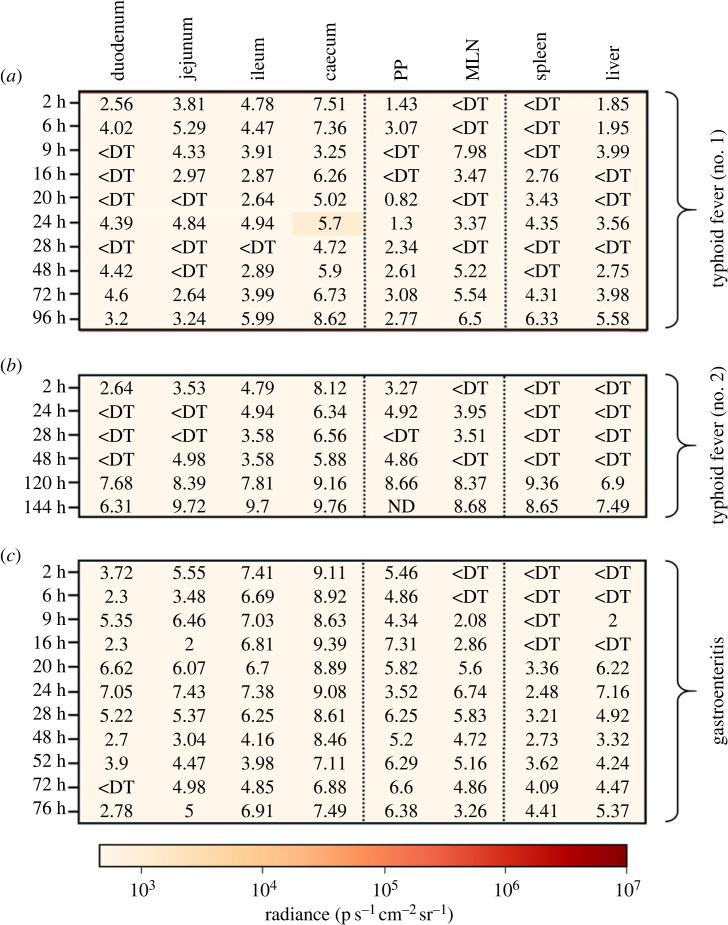
Absolute intensities of bioluminescent signals associated with *rck* transcription. Heat maps representing the luminescence intensities measured in each of the target organs in mouse models (*a* and *b*) of typhoid fever (BALB/c line) or (*c*) of gastroenteritis (BALB/c line pretreated with streptomycin) following oral inoculation of the animals with 5 × 10^8^ CFU of *S*. Typhimurium 14028 P*pefI-srgC*::*lux* strain. The observation times post-infection are specified on the left of the maps, and the bacterial loads expressed in log CFU per gram are indicated on the maps. For the PP, the bacterial loads are expressed in log CFU per PP. ND means not determined and <DT corresponds to organs containing no *Salmonella* or a bacterial load below the detection threshold. PP: ileal Peyer's patches; MLN: mesenteric lymph nodes.

**Figure 5.**
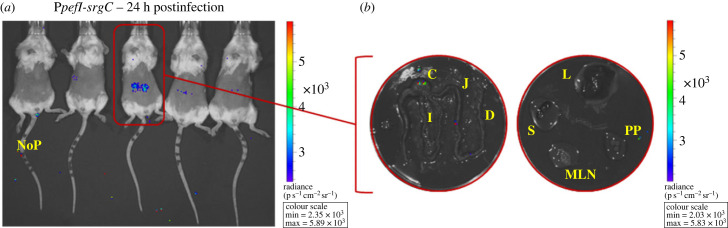
*In vivo* imaging of infection with a *Salmonella* strain expressing the *lux* operon under the control of the P*pefI-srgC* promoter in a murine typhoid fever model. BALB/c mice were orally inoculated with 5 × 10^8^ CFU of *S*. Typhimurium 14028 carrying the mock fusion (no promoter: NoP::*lux*) or the P*pefI-srgC*::*lux* transcriptional fusion. (*a*) One control mouse (NoP) and four P*pefI-srgC*::*lux* mice were imaged at 24 h post-infection with the IVIS Spectrum. The colour scale shows relative signal intensity or radiance (expressed in p s^−^^1^ cm^−^^2^ sr^−^^1^), red being the most intense and blue the least intense. (*b*) Isolated intestine and organs from the positive P*pefI-srgC*::*lux* mouse were imaged at 24 h post-infection. C, caecum; D, duodenum; I, ileum; J, jejunum; L, liver; MLN, mesenteric lymph nodes; PP, ileal Peyer's patches; S, spleen.

In the mouse model of systemic infection (typhoid fever model), we were able to observe one unique bioluminescent signal of low intensity in the caecum (1161 p s^−1^ cm^−2^ sr^−1^) but higher than the positivity threshold (1139 p s^−^^1^ cm^−2^ sr^−1^; electronic supplementary material, figure S2) in our first experiment, suggesting the transcription of the *pefI-srgC* operon within the caecal compartment 24 h post-infection (figure 4*a*). The images of whole body of living animals and of isolated organs obtained for this positive mouse are given in figure 5. In a second experiment performed to focus on later time points of infection, we were not able to detect a bioluminescent signal 24 h post-infection or any other time points despite caecal bacterial loads similar, or even greater than that detected in the first experiment (figure 4*b*). It therefore remains risky, based on these results, to conclude on a real activity of the P*pefI-srgC* promoter region. Similarly, none of the bioluminescent signals measured during the experiment conducted in the gastroenteritis model turned out to be more intense than the positivity threshold set for each organ studied (figure 4*c*). These results suggest that none of the two known promoters of the *pefI-srgC* operon are activated in the typhoid-like and gastroenteritis mouse models.

### 2.5. *pagN* is transcribed within all sections of the intestine, as well as in the MLN, the spleen, and the liver

**Figure 6.**
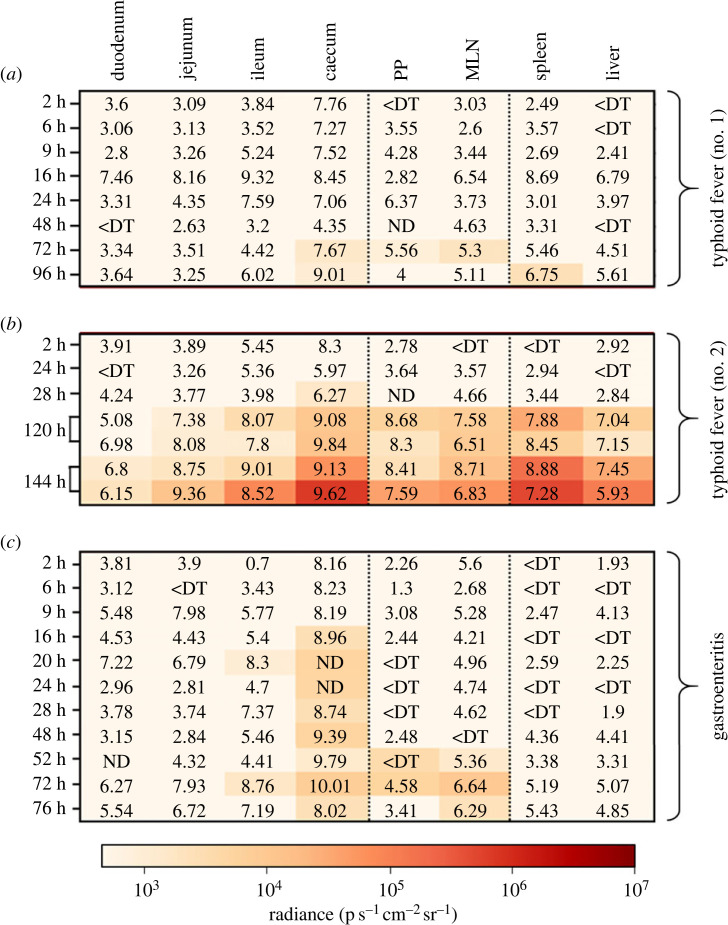
Absolute intensities of bioluminescent signals associated with *pagN* transcription. Heat maps representing the luminescence intensities measured in each of the target organs in mouse models (*a* and *b*) of typhoid fever (BALB/c line) or (*c*) of gastroenteritis (BALB/c line pretreated with streptomycin) following inoculation of the animals with 5 × 10^8^ CFU of *S*. Typhimurium 14028 P*pagN*::*lux* strain. The observation times post-infection are specified on the left of the maps, and the bacterial loads expressed in log CFU per gram are indicated on the maps. For the PP, the bacterial loads are expressed in log CFU per PP. ND means not determined and <DT corresponds to organs containing no *Salmonella* or a bacterial load below the detection threshold. PP: ileal Peyer's patches; MLN: mesenteric lymph nodes.

A first kinetics experiment in the typhoid fever model extending from 2 h to 96 h post-infection supports the postulates of a role of PagN before and after crossing the epithelial barrier. Indeed, we were able to measure bioluminescent signals reporting the transcription of the *pagN* gene within the caecum, and within the ileal Peyer's patches and the MLN 72 h post-infection, and within the spleen 24 h later (figure 6*a*). Figure 6*b* supports the identification of the caecum as a key intestinal site for *pagN* transcription, since signals could be observed in this organ in a second experiment as early as 48 h post-infection and until the end of the experiment. This observation might not strictly be dependent on the bacterial loads in this organ, since the bacterial loads measured at earlier points were higher (i.e. 2 h post-infection: 8.3 log CFU g^−1^) or comparable (24 h post-infection: 5.97 log CFU g^−1^) to that measured at 48 h (i.e. 6.27 log CFU g^−1^), thus suggesting that like for T3SS-1, other factors, probably environmental factors, influence the transcription of *pagN* in the caecum. Following this, we were able to observe an increase in the number of positive intestinal sections, probably due to the evolution of the bacterial loads. As observed at 72 and 96 h post-infection in the first experiment, signals were measured within the gut-associated lymphoid tissues and the spleen at 120 h and 144 h post-infection, but also in the liver (figure 6*b*) thus confirming the transcription of *pagN* in these organs.

**Figure 7.**
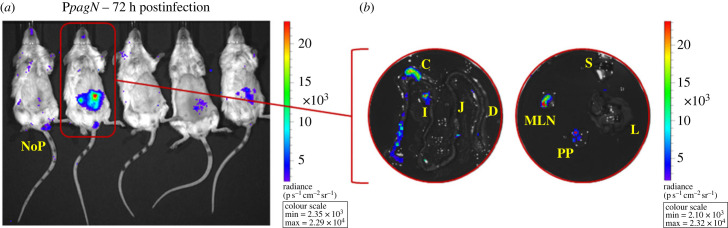
*In vivo* imaging of infection with a *Salmonella* strain expressing the *lux* operon under the control of the P*pagN* promoter in a murine gastroenteritis model. BALB/c mice pretreated with streptomycin were orally inoculated with 5 × 10^8^ CFU of *S*. Typhimurium 14028 carrying the mock fusion (no promoter: NoP::*lux*) or the P*pagN*::*lux* transcriptional fusion. (*a*) One control mouse (NoP) and four P*pagN*::*lux* mice were imaged at 72 h post-infection with the IVIS Spectrum. The colour scale shows relative signal intensity or radiance (expressed in p s^−^^1^ cm^−^^2^ sr^−^^1^), red being the most intense and blue the least intense. (*b*) Isolated intestine and organs from one P*pagN*::*lux* mouse were imaged at 72 h post-infection. C, caecum; D, duodenum; I, ileum; J, jejunum; L, liver; MLN, mesenteric lymph nodes; PP, ileal Peyer's patches; S, spleen.

In the streptomycin-pretreated mouse model, *pagN* transcription-reporting signals were not detected until 16 h post-infection within the caecal compartment, despite comparable bacterial loads at 2 h, 6 h, 9 h and 16 h post-infection (figure 6*c*). This suggest, here again, that our detection capacity is not solely dependent of the number of resident bacteria but would rather result from an evolution of the environment encountered by the pathogen in this organ. Signals were also detected within highly-colonized ileums at 20 h and 72 h post-infection (i.e. 8.3 and 8.76 log CFU g^−1^ respectively) supporting that, as observed in the typhoid-like model, the expression of the virulence factor could also take place within this organ. Similarly, ileal Peyer's patches and MLN were also signal sources, at least 52 h and 72 h post-infection for Peyer's patches, and 52 h, 72 h and 76 h post-infection for MLN (figures 6*c* and 7).

Altogether, these results demonstrate that *pagN* is mainly transcribed in the caecum in the intestine, but also in lymphoid tissues regardless of the mouse model used and in systemic organs in the typhoid-like model.

## 3. Discussion

Independently of the pathology induced by the infecting strain, the infectious process of *Salmonella* requires the invasion of host cells. This key step can be achieved passively, as is the case when interacting with phagocytic cells, or actively using specific factors responsible, among other things, for remodelling of the targeted cell cytoskeleton. The operating principles of the three already known entry factors, namely the T3SS-1 and the OMPs Rck and PagN, are relatively well characterized *in vitro*. However, their respective role in the pathogenesis of *Salmonella* is still poorly understood, especially that of the non-canonical entry factors Rck and PagN. Here, we committed ourselves to identify the anatomical sites of transcription of the genes encoding these factors in relation to the infection kinetics and to assess whether they differ depending on the disease caused by *S.* Typhimurium.

We failed to establish with confidence a transcriptional profile for the *pefI-srgC* operon, and consequently for the *rck* ORF, during mouse infection. This result is somewhat surprising regarding recent findings, demonstrating the presence of molecules of the AHL family recognized as activators of the SdiA regulator in *S*. Typhimurium (i.e. C7-HSL, 3OH-C8-HSL) within the blood, liver and caecal contents of SPF mice [37,38]. However, it remains in agreement with the observations made by Ahmer's Lab, which did not identify SdiA activity or Rck production during infection of mice except when the said-animals were also hosting AHLs-producing bacteria (e.g. *Yersinia enterocolitica, Aeromonas hydrophila*) [29,39]. Two non-exclusive explanations can be proposed. The first would be that the AHLs-producing bacteria were present at a too low concentration (or even absent) to produce enough AHLs capable of inducing the promoter of the P*pefI-srgC*::*lux* fusion. The second would be that these bacteria produce AHLs only under specific conditions not encountered in the context of our experiments. Xue *et al*., for example, demonstrated that *Citrobacter rodentium* infection of SPF C57BL/6 mice leads to an increase of the level of AHLs produced by the gut microbiota community [38].

Additionally, we cannot exclude that the lack of luminescent signals could also be due to the inherent weakness of the P*pefI-srgC* promoter. It can be observed, from the *in vitro* validation experiments of the transcriptional fusions (figure 1), that the emission intensity of the P*pefI-srgC*::*lux* fusion is substantially lower than those of the two other fusions used for this study. Indeed, while under the optimal conditions specific to the induction of the expression of T3SS-1 and PagN, we reached relative luminescence intensities of around 200 000 and 40 000 RLU respectively, we barely peaked at around 10 000 RLU for the P*pefI-srgC*::*lux* fusion under the optimal known conditions for its activation. Another explanation, which does not rule out the first one, could be that our single-copy chromosomally-encoded transcriptional fusion does not reproduce well the transcription of the virulence plasmid-encoded *pefI-srgC* operon. Indeed, Sanchez-Romero *et al.* recently demonstrated that the copy number of the *S.* Typhimurium virulence plasmid could be heterogeneous within a population, ranging from 1 to 8 copies depending on the growth phase [40].

Altogether, our results and those of B. Ahmer's lab are not in favour of a production of the *pefI-srgC* operon encoded products in mice due to a rare and/or too low production of AHLs by the mouse intestinal microbiota. By contrast, when produced, the proteins encoded by this operon play an important role in the intestine of mice [29], thus asking the question in which animal species AHLs production could be sufficient to activate the SdiA regulon. Several reports established, through for example genomics and metagenomic approaches, the presence of bacteria producing AHLs in the digestive tract of species such as humans, cattle, pigs or even horses [41,42]. Consequently, it cannot be ruled out that the use of the genes of the *pefI-srgC* operon can be better appreciated in another host. However, while our approach of *in vivo* live imaging could easily be adapted to other small animals (i.e. chicks, guinea pigs, rabbits, etc.), such a hypothesis would be more complicated to verify experimentally, using this approach, on wider species.

The regulation scheme of T3SS-1 expression is very complex and integrates more than 20 different regulators responding to numerous environmental stimuli. We found a transcription of the *inv/spa* operon in the intestine, a logical expectation given the environment encountered in this organ (adequate osmolarity, low O_2_ concentration, etc.) and the roles previously described of T3SS-1 in epithelial cell invasion and induction of the intestinal inflammatory response [35]. In the typhoid-like model, luminescent signals were detected in the intestine only at the earlier and later stages of the infection while a detection all along the kinetics was observed in the *Salmonella*-induced colitis model. Besides the simple hypothesis of a bacterial load-dependent detection limit, one can conceive that the induction of the inflammation by *Salmonella* and/or the alteration of the microbiota induced by the streptomycin pretreatment could favour the expression of the T3SS-1 through one or several regulatory pathways of the apparatus. However, the environmental components potentially responsible for this different expression remain to be identified. One hypothesis, for example, is that the alteration of the different microbial populations resulting from the antibiotic treatment could lead to a modification of the relative abundances of the different short-chain fatty acids (SCFAs; e.g. acetate, propionate, butyrate) producers within the intestinal lumen as SCFA were shown to modulate the expression of the T3SS-1 through BarA/SirA, HilE, Rcs regulators [43,44]. Outside the intestine, we highlighted the transcription of the operon by MLN-residing bacteria, as well as by those residing within deep-lying organs (spleen and liver in the classic systemic infection model, liver only in the gastroenteritis model). Our observations in the MLN are consistent with data from the literature, notably those published by Giacomodonato *et al.* describing a translocating activity of the T3SS-1 within this tissue [45]. Transcription of the *inv/spa* operon in the spleen and liver is more surprising, especially when considering the work of Gong *et al.* which describes, following infection by the oral or intraperitoneal route, a heterogeneous expression of different components of the secretory apparatus in these organs. Notably, the authors report that they were unable to detect the expression of proteins encoded by the *inv/spa* operon, namely InvJ within the liver [17] and SpaO within the spleen [18]. Although our results do not make it possible to specify whether the T3SS-1 is active in these organs, they clearly show that the *inv/spa* operon is transcribed within the spleen and liver, even if the specific environmental conditions inducing this activation remain to be identified.

Experiments involving the *pagN*-transcription reporting fusion have shown that the gene is transcribed within the caecum independently of the inflammatory context, but also, following the extra-intestinal spread of the pathogen within the reticuloendothelial system. Our observation of an intestinal transcription of the gene suggests a role for PagN in this organ, which remains consistent with the work of Yang *et al.* [24]. Following a phenotypic characterization of a *ΔpagN* strain in the murine colitis model, the authors showed that the deletion of this gene led to an attenuation of the clinical signs of the infection in the intestine (altered epithelium, recruitment of polymorphonuclear granulocytes, etc.) compared to its parent strain 2 days post-infection [24]. A parameter to consider in this study is that the approach used in our study does not make it possible to discriminate between signals of extracellular and intracellular origin. It is known that the main regulatory system of *pagN*, the PhoP/PhoQ system, is induced in bacteria dwelling in the SCV in response to the decrease in pH and the deprivation of divalent cations [46]. The interest of such an intracellular expression site for an entry factor remains to be determined. An extracellular transcription of *pagN* is also strongly supported by a recombination-based gene expression analysis performed *in vivo.* In this study, the PhoP/PhoQ two-component system was shown, through the *pagP* gene, to be activated in luminal bacteria [22]. This idea is also supported by the work of Wilson *et al.* which have demonstrated, in a 3D organoids model infected with a strain restricted to the luminal compartment, that the invalidation of *phoP* is harmful in this compartment in the presence of alpha-defensins [23]. It will be important in future studies to determine whether the transcriptional signals associated with *pagN,* that we detected in this study, are of intracellular or extracellular origin. Similarly for the T3SS-1, it is now well established that, in addition to its essential function in cell invasion, this secretion system plays an important role in the establishment of the cytosolic replication niche of *Salmonella* [47,48]. According to data generated both *in vitro* and *in vivo*, some cell types such as macrophages and fibroblasts seem not to be permissive to this phenomenon. On the other hand, the epithelial cells, abundant within the intestinal tissues identified as transcription sites in our study, were among those displaying a favourable cytosolic environment for this intracellular replication *in vitro*, which would constitute a driving force in the supply of the luminal bacterial pool [19,47,49,50]. There again, further studies are needed to determine the origin of the P*invF*^ON^ populations which could, eventually, clarify the impact of the factor on *Salmonella* pathogenesis. This would be particularly interesting for systemic organs, where luminescent signals were detected, as little is known about the role of the T3SS-1 in host cell invasion and/or cytosolic hyper-replication in these organs.

To conclude, this work has led to the establishment of the spatio-temporal transcription kinetics of genes encoding two of the three entry factors of *Salmonella* in mice. Both are transcribed in the intestine and in the lymphoid-associated tissues. The caecum is the main site of their transcription regardless of the pathological model used, suggesting that the T3SS-1 and PagN could simultaneously mediate *Salmonella* cell invasion in these organs. Whether they mediate invasion of the same cell types or not remains to be determined. By contrast, we observed a different expression of these factors in the spleen and the liver, where *pagN* is highly transcribed in the typhoid-like model at late time points contrary to the *inv/spa* operon that appears more sporadically transcribed, thus suggesting a more important role of PagN than the T3SS-1 during systemic infection. Future work will focus on the identification of cells targeted by these entry factors in the different organs and on the subcellular localization of their expression. These studies should overall allow a better understanding of the respective roles of these virulence factors in the pathogenesis of *Salmonella*.

## 4. Material and methods

### 4.1. Bacterial strains and plasmids

Bacterial strains and plasmids used in this study are listed in table 1. The strains were stored in 25% glycerol at −80°C, and cultured at 37°C in TSB medium, unless otherwise stated, under antibiotic selection when necessary.

**Table 1.** Strains and plasmids used in this study. Cb^r^: carbenicillin resistance; Kan^r^: kanamycin resistance; Cm^r^: chloramphenicol resistance.

strain or plasmid	relevant characteristic(s)	source or reference
strains		
14028	*S. enterica* subsp. *enterica* ser. Typhimurium wild-type strain	American Type Culture Collection
*att*Tn7-NoP::*luxCDABE*	promoterless transcriptional *lux* fusion inserted in the *att*Tn7 site of strain 14028 (Cm^r^)	[51]
*att*Tn7-sig70c35::*luxCDABE*	transcriptional fusion sig70c35::*lux* inserted in the *att*Tn7 site of strain 14028 (Cm^r^)	[51]
*att*Tn7-P*pefI-srgC*::*luxCDABE*	transcriptional fusion P*pefI-srgC*::*lux* inserted in the *att*Tn7 site of strain 14028 (Cm^r^)	this work
*att*Tn7-P*pagN*::*luxCDABE*	transcriptional fusion P*pagN*::*lux* inserted in the *att*Tn7 site of strain 14028 (Cm^r^)	this work
*att*Tn7-P*invF*:::*luxCDABE*	transcriptional fusion P*invF*::*lux* inserted in the *att*Tn7 site of strain 14028 (Cm^r^)	this work
MC1061	*E. coli hsdR mcrB araD*139 *Δ*(*araABC-leu*)7679 *ΔlacX*74 *galU galK rpsL thi*	[52]
CC118	*E. coli Δlac*X74 *galE galK phoA*20 *thi*, r*psE rpoB argE* (Am) *recA*1(*λpir*)	[53]
plasmids		
pCS26-sig70c35::*luxCDABE*	plasmid-borne construction allowing constitutive expression of the *luxCDABE* luciferase system (Cm^r^)	[33]
pUC18R6K-mini-Tn7T-PacI	delivery vector with a *λpir*-dependent replication (Cb^r^)	[33]
pHSG415-*tnsABCD*	helper plasmid with temperature-sensitive replication (Cb^r^)	[33]
pACYC177	cloning vector (Cb^r^, Kan^r^)	[54]
pACSdiA	pACYC177 containing *S*. Typhimurium 14028 *sdiA* ORF and its RBS (Cb^r^)	[26]

### 4.2. Generation of reporter fusions

The chromosomally-encoded transcriptional fusions were designed as described by Shivak *et al.* [33]. To simplify the writing, the *luxCDABE* operon will be abbreviated as ‘*lux*’ in the names of all plasmids and strains throughout the manuscript. Briefly, promoter regions of entry factors (P*pagN*, P*pefI-srgC*, and P*invF*) were amplified by PCR from the genome of *S.* Typhimurium 14028 (GenBank accession number CP001363) using the appropriate primers listed in electronic supplementary material, table S1. A schematic representation of each targeted region is shown in figure 1*a*. The subsequent PCR products were restricted with XhoI and BamHI enzymes, then ligated within XhoI/BamHI-restricted pCS26-sig70c35::*luxCDABE* plasmid and used to transform chemically competent *E. coli* MC1061 bacteria.

Recombinant bacteria were selected on TSA plates containing chloramphenicol (30 µg ml^−1^). Plasmid constructions were then checked by PCR using primers pCS26-FWD and pCS26-lux-REV (electronic supplementary material, table S1) and sequencing. pCS26-sig70c35::*luxCDABE* derivatives were then restricted by PacI, ligated within PacI-restricted pUC18R6K-miniTn7T, and used to transform chemically competent *E. coli* CC118 (*λpir*) bacteria to amplify the subsequent pUC18R6K-miniTn7T-*luxCDABE* derivatives. Recombinant bacteria were selected on TSA plates supplemented with chloramphenicol (30 µg ml^−1^) and carbenicillin (100 µg ml^−1^). To ensure homogeneous orientation of the transcriptional fusions within the vector, the resulting plasmids were restricted by BglII. pUC18R6K-miniTn7T-*luxCDABE* derivatives with the correct orientation were then used to transform electrocompetent *S*. Typhimurium 14028 harbouring thermosensitive pHSG415-*tnsABCD*. Homologous recombination of the fusions at the *att*Tn7 site was performed as described by Shivak *et al.* [33]. The resulting clones were checked by PCR using the primers described in electronic supplementary material, table S1 to ensure the correct and homogeneous orientation of the inserts between the strains (electronic supplementary material, figure S1), and then sequenced.

### 4.3. *In vitro* validation of the reporting fusions

Overnight cultures of strains harbouring the chromosomal transcriptional fusions in TSB medium containing 30 µg ml^−1^ chloramphenicol were used to inoculate, at 1 : 100, the appropriate media for each fusion. The resulting suspensions were then dispensed into white-walled 96-well plates (200 µl per well; Corning, no. 3610) in order to perform kinetic measurements of optical density at 600 nm and luminescence intensity at 37°C with shaking in a microplate reader (Infinite M Plex, Tecan). For the P*invF*::*lux* fusion, measures were performed in Lennox broth [32] with a 12-hour kinetics. The strains carrying the P*pefI-srgC*::*lux* or the Psig70c35::*lux* fusion were used as negative and positive controls, respectively. The validation of the P*pefI-srgC*::*lux* fusion was done using the activation conditions previously described [26]. The strain was transformed with a plasmid constitutively expressing SdiA (pACSdiA) or with the empty vector (pACYC177, negative control) and measures of luminescence were performed in TSB medium containing 100 μg ml^−^^1^ carbenicillin and 2 μM N-hexanoyl-l-homoserine lactones (C6-HSL or AHL; Cayman). The P*pagN*::*lux* fusion was verified by using the inducing medium LPM (low phosphate, low magnesium-containing medium; pH 5.8) [55]. Overnight cultures were washed once in ultrapure water and diluted 1 : 50 in LPM or 1 : 100 in Lennox broth (negative control) for kinetic measurements. All assays were performed in quintuplicate from at least three independent bacterial cultures.

### 4.4. *In vivo* mice experiments

The *in vivo* experiments were carried out in strict compliance with French legislation. The protocols for this study have been validated by the French Ministry of National Education, Higher Education and Research under authorization numbers 03749.03 (30 April 2015) and APAFIS no. 23937-202002041548450 v3 (11 May 2020). All animal experimentations were performed in the Infectiology of Farm, Model and Wildlife Animals Facility (PFIE, Centre INRAE Val De Loire: https://doi.org/10.15454/1.5572352821559333E12; member of the National Infrastructure EMERG'IN). Inocula were prepared as previously described [51]. The typhoid-like and the gastroenteritis models were used as described [36,56]. Briefly, 6–8 week-old female BALB/c mice were orally inoculated with 5 × 10^8^ CFU of one of the different luminescent *S.* Typhimurium 14028 strains (table 1). In the gastroenteritis model, mice were pretreated with 20 mg streptomycin 24 h before *Salmonella* inoculation. At each time point of interest, mice were anaesthetized (Vetflurane (3%) mixed oxygen (1.5 l min^−1^) gas), and optically imaged using a photon-counting system (IVIS Spectrum, PerkinElmer). One animal per time-point was sacrificed by cervical dislocation, its organs of interest (spleen, MLN, duodenum, ileum, jejunum, caecum, ileal Peyer's patches, and liver) isolated and imaged. In a few cases, two animals were sacrificed at the same time point. Bioluminescence quantification was performed using the Living Image 4.5.5 software (PerkinElmer). Results are expressed in photons per second per square centimetre per steradian (p s^−1^ cm^−2^ sr^−1^), that is, radiance. Then, bacterial loads of the different organs were determined as previously described [51]. Two different schedules were tested in the typhoid fever model in order to assess all the kinetics of *S.* Typhimurium infection.

## Data Availability

Luminescence raw data for all organs have been deposited in the data repository Data INRAE at https://doi.org/10.57745/QLQZ16 [57]. Supplementary material is available online [58].
